# Ten Steps to Conducting a Large, Multi-Site, Longitudinal Investigation of Language and Reading in Young Children

**DOI:** 10.3389/fpsyg.2016.00419

**Published:** 2016-03-30

**Authors:** Language and Reading Research Consortium (LAARC), Kelly Farquharson, Kimberly A. Murphy

**Affiliations:** ^1^Department of Communication Sciences and Disorders, Emerson CollegeBoston, MA, USA; ^2^Crane Center for Early Childhood Research and Policy, The Ohio State UniversityColumbus, OH, USA

**Keywords:** longitudinal studies, reading development, protocols, reading comprehension, language development

## Abstract

**Purpose:** This paper describes methodological procedures involving execution of a large-scale, multi-site longitudinal study of language and reading comprehension in young children. Researchers in the Language and Reading Research Consortium (LARRC) developed and implemented these procedures to ensure data integrity across multiple sites, schools, and grades. Specifically, major features of our approach, as well as lessons learned, are summarized in 10 steps essential for successful completion of a large-scale longitudinal investigation in early grades.

**Method**: Over 5 years, children in preschool through third grade were administered a battery of 35 higher- and lower-level language, listening, and reading comprehension measures (RCM). Data were collected from children, their teachers, and their parents/guardians at four sites across the United States. Substantial and rigorous effort was aimed toward maintaining consistency in processes and data management across sites for children, assessors, and staff.

**Conclusion**: With appropriate planning, flexibility, and communication strategies in place, LARRC developed and executed a successful multi-site longitudinal research study that will meet its goal of investigating the contribution and role of language skills in the development of children's listening and reading comprehension. Through dissemination of our design strategies and lessons learned, research teams embarking on similar endeavors can be better equipped to anticipate the challenges.

In 2010, the United States (U.S.) Department of Education Institute for Education Sciences (IES) embarked on a large-scale and rigorous initiative aimed at improving reading comprehension for all students. This investment, *Reading for Understanding* (RFU; see Douglas and Albro, 2014, for complete details), is one of the nation's largest initiatives of its kind and is in response to evidence of continued poor academic performance among American children on such indicators as the National Assessment of Educational Progress (NAEP). For example, recent NAEP data revealed that 33% of fourth-grade children and 25% of eighth-grade children cannot read at the basic level and consequently cannot understand what they read (IES, 2009). In response to the IES initiative, four core projects were charged with: (1) identifying underlying processes related to reading comprehension that are malleable and thus present reasonable targets for intervention and (2) developing and evaluating interventions based on these malleable processes. The purpose of this descriptive paper is to provide a methodological overview of the conceptual framework, research questions, research design, measures, and data processing for one of the four core projects within RFU, the Language and Reading Research Consortium (LARRC), which focuses on early childhood, defined for our purposes as preschool (PK) through grade 3 (G3). Specifically, the LARRC multi-site longitudinal study sought to rigorously investigate the contribution and role of higher- and lower-level language skills to the development of children's listening and reading comprehension.

Although there has been some effort made by research teams to share their design challenges and lessons learned during implementation of complex research studies (e.g., Wagner et al., 1991), design details and recommendations are often difficult to locate outside of the brief methods sections typically available in published manuscripts. Consequently, one goal of the present paper is to contribute to the literature by describing the complexities of large-scale and multi-site research projects. Similar to recent reports (McDonald et al., 2006; Rudd and Johnson, 2008; Ong-Dean et al., 2011), a second goal of this paper is to outline methodological “lessons learned” during the process of executing a rigorous longitudinal investigation. Specifically, we have presented 10 steps that can be taken to carefully design and coordinate this large project. Table 1 provides a list of these 10 steps. In detailing these steps, we hope not only to respond to calls for transparent disclosure of large-scale, multi-site research protocols (Sterba et al., 2011) but also provide information to assist in the planning and execution of future studies similar in complexity.

**Table 1:** **Ten steps for completing a longitudinal investigation**.

**Step number**	**Step title**
1	Build a collaborative team and set a long-term plan
2	Develop a strong theoretical framework to support research questions
3	Design a comprehensive study which maps onto study aims
4	Determine the sample and develop a recruitment plan
5	Select and/or Develop measures
6	Train assessors and develop data collection procedures
7	Commence data collection
8	Develop a plan and team for data scoring
9	Develop and implement a strong plan for data management
10	Prepare for frequent and thorough communication, flexibility, and challenges

## Step one: Build a collaborative team and set a long-term plan

In order to build a multi-site longitudinal study, we created a team that would appropriately support our goal of investigating listening and reading comprehension in PK to G3. To that end, LARRC is a multi-university network of interdisciplinary researchers located at four universities across the United States (Arizona State University [ASU], the Ohio State University [OSU], University of Kansas [KU], and University of Nebraska-Lincoln [UNL^1^]) and also Lancaster University in the United Kingdom (Lancaster University participated in measure development, research design, and overall planning but not data collection). Collectively, LARRC researchers exhibit expertise in speech language pathology, developmental and experimental psychology, reading research, intervention design, professional development, and quantitative methodologies. Of this LARRC group, several scientists were the principal investigators at each site, whereas others served on LARRC review committees to advise and guide the team during measure development and selection and to ensure that all study plans adhered to rigorous data-driven methodology. Additionally, study implementation was supervised by the funding agency during quarterly RFU team meetings in Washington, DC (Douglas and Albro, 2014).

LARRC researchers developed a long-term plan for answering the call of the RFU initiative. Specifically, this plan included a collection of three studies, to be completed over the course of 5 years, moving toward the long-range goal of improving children's language abilities, background knowledge, and listening and reading comprehension. Study 1 was a longitudinal research study, described in detail in the remainder of this document, concerning the basic cognitive processes underlying reading comprehension and the development of reading comprehension from PK through G3 in English speaking children. The purpose of Study 2 was to define and develop two different instantiations of a language-based comprehension curriculum supplement for each of grades PK to G3. Study 3 involved efficacy trials of the language-based comprehension curriculum supplement through a randomized controlled trial for PK to G3. For all three studies, a companion Spanish-English bilingual study followed similar procedures. Studies 2, 3, and the companion bilingual study will not be discussed here (but see LARRC, in press for complete information on the bilingual companion study and Study 2). The current paper is a methodological review of LARRC Study 1, a multi-site, multi-cohort longitudinal investigation into the contribution and role of higher- and lower-level language skills to the development of English-speaking children's listening and reading comprehension.

### Lessons learned

The varied experiences of the LARRC research team created a strong and diverse foundation upon which to build this study. Frequent communication, in the form of in-person, on-line, or teleconference, served as frequent opportunities to discuss the overall goal of this large project. Many team members held particular expertise without which the overall design could not have been achieved. For example, investigators not only held different content expertise that influenced the design (e.g., decisions to assess not only child factors but also home and classroom environments) but also held differing expertise in various designs and methods (e.g., longitudinal designs, cross-sectional designs, lab- and field-based data collection activities). We expand on how these lessons affected study design in Step 3.

## Step two: Develop a strong theoretical framework to support research questions

Building on the aforementioned strengths of the multi-disciplinary team, we sought to focus our theoretical framework on specific content areas of each team member. Our theoretical framework emphasized language as a developmental construct central to our activities and to our focus on the language bases of reading comprehension. This framework built on prominent models of reading comprehension, all of which identify language as a critical contributing factor. For example, the Simple View of Reading (Gough and Tunmer, 1986; Hoover and Gough, 1990; see also Joshi and Aaron, 2000, 2012) posits that skilled reading comprehension is the product of word decoding and listening comprehension. McCardle et al. (2001) further discuss a model of reading development, which posits that both word recognition and language comprehension skills are essential for reading comprehension; knowledge of vocabulary, language structures, and verbal reasoning are also contributing language components. Additional work has expanded these theories to identify the particular importance of both higher-level (e.g., inferencing and integrating, comprehension monitoring, text structure knowledge) and lower-level (e.g., vocabulary, syntax, morphology) language in skilled listening and reading comprehension (Perfetti et al., 2005; Perfetti, 2007).

As a recent example of this expansion, models of reading proposed by Perfetti (2007), Perfetti et al. (2005) highlight the fundamental role of a well-organized lexicon with highly-specified representations of word forms and meanings. Such verbal efficiency, in terms of lower-level language, affords opportunities to engage higher-level language resources used to construct meaning from text. These higher-level skills are crucial for creating the mental models of text that allow for successful reading comprehension (see also McNamara and Magliano, 2009, for review of other models of reading comprehension emphasizing higher-level skills).

Given that language skills make a substantial contribution to one's ability to read for meaning, LARRC researchers hypothesized that early interventions focused on the language bases of reading comprehension may have the greatest impact on future reading comprehension and also reduce risks for comprehension-specific reading difficulties. Considering our theoretical framework and this hypothesis, we chose a battery of assessments that would allow for comprehensive examination of language and reading skills in our longitudinal work. Our study design and comprehensive assessment battery supports critical examination of theoretically important higher- and lower-level language skills from early childhood, when children are not yet reading or just beginning to emerge as readers, to the period of time when children are able to read for meaning. Data from this longitudinal study will be used to test whether and how these specific component language contributions vary developmentally over time and how these trajectories may be influenced by individual, environmental, and classroom characteristics.

### Lessons learned

We were fortunate to have a team of diverse researchers who were well-versed in the aforementioned theories. As such, each investigator was able to recommend assessment tools that would sensitively capture the constructs reflected in each complementary theory. Although this created a significant number of assessments to administer, it also created a rich dataset. This dataset has already been used to explore the relevant theories in question (Language and Reading Research Consortium (LARRC), 2015a,b, in review; Murphy et al., in press). However, to attempt to streamline assessment administration, some measures were ultimately omitted from the test batteries. For instance, measures of spelling or detailed orthographic knowledge were omitted but would have created a robust opportunity to deeply explore the Lexical Quality Hypothesis (Perfetti, 2007). Ultimately, our team felt strongly that we were robustly examining the constructs of interest and, certainly, we have substantial data to explore multiple theoretically driven questions.

## Step three: Design a comprehensive study that maps onto study aims

Once our theoretical framework was established, the LARRC team began to design a longitudinal study that would allow for a comprehensive examination of listening and reading comprehension skills in children PK-G3. Our approach to the research design and analysis process has been one of transparency and collaboration throughout, necessitated by our participation in the larger RFU network, the need to coordinate efforts among diverse researchers at geographically dispersed universities, and our desire to meet the highest standards of rigor and quality in educational research. With respect to the latter, the National Research Council (2002) reported on six guiding principles that underlie scientific inquiry and contribute to significant advances in scientific knowledge. These include posing significant questions that can be empirically investigated, link research to relevant theory, use methods that allow direct investigation of the question, provide a coherent and explicit chain of reasoning, replicate across studies, and disclose research to professional scrutiny and critique.

### Lessons learned

As we designed the longitudinal study, we learned a number of important lessons. First and foremost, and as will be echoed in Step 10, communication among investigators was key. When initially designing the study, investigators met face-to-face in full-day meetings to discuss research questions and options for addressing these. This face-to-face communication not only allowed for multiple design options to be vetted but also commenced relationship building among investigators that was foundational for the continued success of the project. Second, we found it critical to involve all investigators in the initial design phase. This obliged the team to consider a full range of design and data collection options, which we believe not only improved our final design but also set the tone for project collaboration, in that all investigators' ideas and expertise were welcomed and respected. Finally, the inclusion of methodologists in all design conversations was imperative. Methodologists were involved as full team members, as opposed to consultants, for the entire duration of the project. This ensured that our design was appropriate for planned analyses from its inception and also helped alleviate potential methodological flaws as the project progressed.

## Step four: Determine the sample and develop a recruitment plan

The LARRC study features a complex sampling design, and we have aligned our sampling methods with established principles of high-quality scientific research (NRC, 2002). Despite the use of a non-probability sample, the composition of our sample spans PK through G3 and intentionally provides an appropriate model of the general U.S. population for these grades with some limitations as described below. We established cohorts at each grade, PK through G3, at four data collection sites (ASU, KU, OSU, and UNL). Separate approval was obtained from each of the sites' Intuitional Review Board (IRB). Each cohort was followed and assessed annually until reaching G3. This multi-cohort longitudinal design affords both cross-sectional and longitudinal analyses (see Table 2 for a depiction of this process). With four data collection sites, consistency in sampling procedures was paramount. Our primary intent was to design a logistically feasible study with adequate power to detect the relations of interest; we also placed specific emphasis on retention of the longitudinal PK cohort, which would be followed for the entire study duration of 5 years. The multi-site cohort design supported our child enrollment goals in terms of representativeness of the U.S. PK to G3 population, allowed us to enroll and assess a large number of children in a very narrow time window, and accommodated natural variability in classrooms within- and between-schools. A-priori power analyses (with power ≥ 0.80) for the multiple cohort design and with adjustments for approximately 20% attrition (US Department of Education, Institute for Educational Sciences [IES], 2013) indicated a targeted sample size goal of 400 PK children and 120 children in each of K-G3 across all sites;^2^ these sample size goals included follow-up through the 5-year study period up to and including G3. At year 1, each data collection site was responsible for recruitment of one-fourth of the total child sample with corresponding retention goals for follow-up assessments in subsequent years. Table 2 provides the overall enrollment goals for the number of children assessed per year, by site and across all sites. To achieve these child enrollment goals, sites proceeded to first recruit local schools and teachers, and then recruit children from the classrooms of participating teachers.

**Table 2:** **Targeted number of children assessed/followed per year, per site**.

**GRADE**	**PK**		**K**		**G1**		**G2**		**G3**	***N* (site)**	***N* (total)**
Year 1	PK = 100		K = 30		G1 = 30		G2 = 30		G3 = 30	220	880
Year 2			K = 100		G1 = 30	↘	G2 = 30	↘	G3 = 30	190	760
Year 3		↘		↘	G1 = 100		G2 = 30	↘	G3 = 30	160	640
Year 4				↘		↘	G2 = 100		G3 = 30	130	520
Year 5						↘		↘	G3 = 100	100	400
								↘			

*The number listed in the Year 1 row is the initial number of participants recruited per grade, per site. Following Year 1 diagonally per grade illustrates the number of participants who were followed longitudinally from the original sample (e.g., In Year 1, 100 preschoolers were recruited at each site and all 100 preschoolers will be followed longitudinally at each site for a total of 5 years). PK, preschool; K, kindergarten; G1, Grade 1; G2, Grade 2; G3, Grade 3. N (site) refers to the total number of children recruited per grade per site; N (total) refers to the total number of children recruited per grade across all sites*.

### School and teacher recruitment

We did not place any restrictions on the number of schools to be recruited, or the number of teachers per school who would participate; sites managed their own recruitment processes such that a sufficient number of schools and teachers were recruited to obtain the necessary child enrollment. Generally, districts and schools were recruited prior to recruiting and consenting individual teachers. At each site, school districts and/or individual schools were selected primarily because of size and diversity of the student populations, as well as willingness to work with LARRC researchers. Several sites had previously established relationships with local school districts and were able to more quickly recruit based on these partnerships. Based on recommendations from district personnel, additional schools who had not previously partnered with the research teams were contacted and invited to participate.

Once permission was obtained from district and/or individual school leaders, all teachers in the relevant grades were invited to participate. Research staff or school personnel explained the study to classroom teachers via open or individual informational meetings and provided teacher consent packets detailing the study and the rights and responsibilities of researchers, teachers, students, and families. Teachers willing to (a) facilitate child recruitment in their classrooms, (b) complete questionnaires and child indirect assessments, and (c) participate in classroom observations were consented into year 1 of the study. In subsequent years, children matriculated into additional teachers' classrooms. These teachers were similarly approached by research staff and consented. All participating teachers received incentives for their participation; these incentives were tied to the research activities performed and varied by site, in accordance with individual Internal Review Board and school district requirements.

### Child recruitment

In year 1, children were recruited from the classrooms of participating teachers. Teachers sent recruitment packets home with every child in their classrooms. These recruitment packets contained literature on LARRC, a caregiver permission form, a family screener questionnaire, and a return envelope. Caregivers could return the forms directly back to the teacher or mail them to the LARRC project. Teachers were also asked to complete brief screener questionnaires for all children who returned consent forms. This process was used until sites recruited up to 30 but no more than 50 consented children from each grade K-G3, and 110 PK consented children. Such over-recruiting was utilized to compensate for attrition through study withdraw or family mobility.

Research staff reviewed family screener and teacher screener questionnaires to ensure that consented children could appropriately participate in assessments selected for the general sample. This included being rated as understanding and speaking English fluently and not having severe or profound disabilities that greatly affected the child's classroom functioning. In addition, (a) PK children had to be expected to matriculate to K the following academic year, and (b) siblings and twins were excluded from the sample to the extent possible to reduce dependencies in the data (i.e., nesting in families as well as classrooms).

### Child demographics

In the first year of the longitudinal study (baseline) we recruited a total of 915 children in grades PK through G3. Of these, 416 children were in PK, 128 children in K, 125 in G1, and 123 in each G2 and G3 (see Table 3 for demographic breakdown by grade).

**Table 3:** **Selected Baseline (2010) child characteristic for the longitudinal study**.

**Characteristic**	**PK**	**K**	**G1**	**G2**	**G3**
*N*	416	128	125	123	123
Age in months	60.8 (5.4)	72.6 (4.6)	85.0 (4.0)	96.6 (4.5)	109 (4.5)
**INCOME (CATEGORICAL)**					
% < 30 K	12.8	14.8	12.2	17.9	9.6
% 31 K < 60 K	25.3	18.8	23.5	17.1	20.9
% > 60 K	61.9	66.4	64.3	65.0	69.6
% female	41.8	47.7	56.8	48.4	53.7
% white/Caucasian	93.0	83.3	88.9	94.0	85.6
%Hispanic/Latino	6.7	16.1	6.9	8.7	5.1
% Free/Reduced lunch	14.6	21.8	15.5	25.6	16.4
% Individualized education plan	14.0	5.0	7.0	6.0	7.0
% English home language	97.6	100	98.3	96.6	95.7
% both parents live with child	84.6	83.3	77.0	71.8	75.2
% with mom ≤ HS/GED	13.5	15.2	11.1	11.2	9.6

*PK, preschool; K, kindergarten; G1, Grade 1; G2, Grade 2; G3, Grade 3. Income is in increments of 30,000 (i.e., 30 K). HS/GED, High School diploma or General Education Development test, which is an equivalent to high school diploma in the U.S*.

### School and teacher demographics

In our first (baseline) year, 69 schools and/or PK centers were recruited from partnering districts across the four data collection sites. Seventy-eight percent were drawn from suburban locations and 22% from urban locations. Of the 69 schools, 29 were PK-only schools/centers, four served PK and K, four served K only, and 32 were elementary schools. For year 1 teacher demographic information, please see Table 4.

**Table 4:** **Baseline teacher characteristics in the longitudinal study**.

**Characteristic**	**PK**	**K**	**G1**	**G2**	**G3**
*N*	86	50	43	37	41
% female	98.8	98.0	95.2	97.1	97.6
% White/Caucasian	96.4	98.0	95.2	91.2	97.5
% Hispanic/Latino	1.2	0	4.8	5.9	0
% < 5 years of experience	37.5	48.9	45.2	45.5	59.0
% Masters' degree or above	35.8	59.1	57.5	73.5	72.5

*PK, preschool; K, kindergarten; G1, Grade 1; G2, Grade 2; G3, Grade 3*.

Across these 69 schools, 258 teachers participated in our study. The participating teachers were predominantly female (98.5%). For the 88 PK teachers, 70% reported that their school/center provided half-day programs only and 22.5% reported having both full- and half-day programs; the remainder were teachers in full-day only centers or schools. For the 49 K teachers, only 2.2% reported teaching in half-day programs, with another 2.2% indicating that their school/center supported both full- and half-day programs. For primary grades, we recruited 43 G1 teachers, 37 G2 teachers, and 41 G3 teachers.

### Lessons learned

Four essential lessons were learned as we recruited our sample. First, the involvement of multiple sites was both beneficial and challenging. The largest benefit was the swift recruitment of almost 1000 children, which likely could not have been accomplished at a single site. At the same time, coordinating among multiple university IRBs was a challenge. Although we had initially intended to use the same wording on recruitment and consent documents, and also provide the same participant incentives across sites, this proved untenable given differences in IRB policies. The geographic locations of our sites (e.g., Kansas, Nebraska, Ohio) also resulted in a sample that was less racially and ethnically diverse than the general U.S. population. Second, we learned the importance of personal relationships when conducting field-based research in schools. Our quick recruitment was greatly aided by the relationships that research teams had previously established with local school personnel. We found it considerably more challenging to gain access in schools where we had no such relationships. In these cases, we found that it often helped if (a) an introduction with the district or building administrator could be arranged by other school-based professionals with whom the research team already had a relationship (e.g., one principal providing an introduction to another), (b) initial meetings were face-to-face rather than via phone or email, and (c) emphasis was placed on the project as a team effort, in terms of the potential benefits for the school partner, flexibility within the prescribed parameters of the research project (e.g., scheduling assessments so as not to conflict with standardized testing or other important dates), and willingness to share and interpret findings. Notably, without personal relationships already established, it was particularly difficult to gain access to those schools serving children at risk for academic difficulties (e.g., due to socioeconomic disadvantage), with research participation understandably not of the highest priority. Third, we learned that willingness to participate in the project shifted over time. This occurred in two key ways. The first was due to the timing of grant submission vs. actual implementation. We had recruited many partnering schools when the grant was initially submitted, to ensure that the project was feasible and to provide required letters of support with the proposal. However, given the span of time until the project was awarded, circumstances for some of these schools had changed such that they were no longer able to participate and new schools had to be recruited. The second shift in willingness to participate was due to the longitudinal design. Because we followed children as they matriculated from grade to grade, teachers in subsequent grades were not necessarily those who had initially “bought in” to the research project. These teachers were often less willing to consent to research activities and alternative arrangements were made (e.g., assessing children outside the school day, not conducting videotaped classroom observations). Fourth, we learned the need to devote sufficient resources for recruitment and retention beyond the initial year of the study. As noted above, although new children were not recruited in subsequent years, new teachers needed to be consented as children matriculated into their classrooms. This was best accomplished when research team members were able to once again make personal visits to schools to share information about the project. Moreover, children needed to be tracked as they moved into not only different classrooms but often different schools, particularly those who began participation during their PK year. This involved substantial time and effort on the part of research staff, to contact parents and school personnel and locate children in the weeks preceding each new school year. In addition, substantial resources were devoted to preventing attrition. This included having a well-maintained project website, periodically requesting updated contact information from parents, sending project emails and newsletters to participants, regularly collecting feedback from parents and school personnel, and mailing thank you and birthday cards to participants.

## Step five: Select and/or develop measures

Our theoretical model and hypotheses regarding the relations between language and reading comprehension guided our measures selection, in addition to other important considerations. Measures had to be appropriate for the age- and grade-range of participating children, with measures assessing each construct from the LARRC theoretical framework included at each grade level. Preference was afforded to measures that spanned PK through G3, in order to maintain consistency, continuity, and equivalency in scores, which in turn, allowed us to examine developmental change over time (i.e., compare language and literacy skills across grades/ages). This principle proved easier to apply for some constructs (e.g., vocabulary) than others (e.g., comprehension monitoring), and some measures (a) had to be modified for use with younger children, (b) could only be administered to children in particular grades, and (c) had to be created specifically for this project, as no established measures existed. Preference was also afforded to measures with established psychometrics. In many cases, use of established measures provided some information allowing comparison to a U.S. normative sample via standard scores or percentile ranks, enhancing generalizability. Measure relevance and administration time also had to be balanced. Finally, we were keen to include multiple measures of key constructs, to afford creation of latent variables and control for measurement error often associated with examination of complex cognitive abilities (Kline, 2004). We considered this particularly important for the measurement of comprehension, as research suggests that reading comprehension measurement may be influenced by different assessment modalities and variability in task demands (e.g., Cutting and Scarborough, 2006). Ultimately, the LARRC assessment battery comprised measures in three broad categories: (a) direct measures, (b) indirect measures, and (c) observational measures. Details concerning the measures are presented in Table 5.

**Table 5:** **Measures by construct**.

**Measure**	**Generalizable/Experimental**	**Direct/Indirect/Observational**	**Grade**
**LANGUAGE SKILLS**			
**Lower-Level Language: Grammar**			
CELF-4 word structure	G	D	PK,K,1-3
CELF-4 recalling sentences	G	D	PK,K,1-3
TEGI screener	G	D	PK, K
TROG-2	G	D	PK,K,1-3
TNL-5 story retell	G	D	PK,K,1-3
Morphological lexical judgment	G	D	PK,K
Wagner morphological derivation task	G	D	K,1-3
**Lower-Level Language: Vocabulary**			
PPVT-4	G	D	PK,K,1-3
EVT-2	G	D	PK,K,1-3
CELF-4 word classes	G	D	PK,K,1-3
TNL- number of different words	G	D	PK,K,1-3
**Higher-Level Language**			
Inference making stories	E	D	PK,K,1-3
Comprehension monitoring: knowledge violations	E	D	PK,K
Comprehension monitoring: inconsistencies	E	D	PK,K,1-3
TNL episodic analysis	G	D	PK,K,1-3
Narrative structure task—picture arrangement	E	D	PK,K,1
Narrative structure task—sentence arrangement	E	D	2-3
**LISTENING AND READING COMPREHENSION**			
**Listening Comprehension**			
CELF 4 understanding spoken paragraphs	G	D	PK,K,1-3
TNL Receptive	G	D	PK,K,1-3
Listening comprehension measure	G	D	PK,K,1-3
**Reading comprehension**			
GMRT	G	D	1-3
Reading comprehension measure	G	D	1-3
WRMT- passage comprehension	G	D	1-3
**WORKING MEMORY AND RELATED COGNITIVE PROCESSES**			
**Working Memory**			
Non-word repetition task	E	D	PK,K,1-3
WJ-III NU numbers reversed	G	D	1-3
Updating task	E	D	PK,K,1-3
WJ-III NU auditory memory	G	D	PK,K,1-3
**Serial Rapid Naming**			
CELF-4 rapid automatic naming	G	D	PK,K,1-3
**Non-verbal Reasoning**			
KBIT 2	G	D	PK,K,1-3
**DECODING ABILITIES AND READING PRECURSORS**			
**Decoding**			
WRMT—word ID	G	D	K, 1-3
WRMT—word attack	G	D	K, 1-3
TOWRE-2—phonetic decoding/sight	G	D	K, 1-3
FAIR— oral reading fluency	G	D	1-3
**Phonological Awareness**			
TOPEL—phonological awareness	G	D	PK,K
**Print Knowledge**			
TOPEL—print knowledge	G	D	PK,K
WRMT—letter ID	G	D	PK,K
**BEHAVIORAL REGULATION AND READING MOTIVATION**			
Elementary reading attitude survey (ERAS)	G	D	1-3
Preschool reading attitude survey (PRAS)	G	D	PK,K
Social skills improvement system (SSIS)	G	I	PK,K,1-3
Strengths and weaknesses of ADHD-symptoms and normal behavior (SWAN)	G	I	PK,K,1-3
Preschool learning behaviors scale (PLBS)	G	I	PK
Learning behaviors scale (LBS)	G	I	K,1-3
**ENVIRONMENTAL ATTRIBUTES**			
**Home Attributes**			
Home literacy environment	G	I	PK,K,1-3
Parent reading belief inventory	G	I	
Confusion, hubbub, and order scale (CHAOS)	G	I	PK,K,1-3
Child sleep habits	G	I	
**Classroom Attributes**			
CLASS	G	O	PK,K,1-3
ISI	G	O	PK,K,1-3
CLOP/ CLEP	E	O	PK,K,1-3
**Teacher Attributes**			
Teacher self-efficacy scale	G	I	PK,K,1-3
Sense of School community	G	I	PK,K,1-3
Modernity scale	G	I	PK,K,1-3
Teaching beliefs and learning styles	G	I	PK,K,1-3
Teacher professional development	G	I	PK,K,1-3

*G, generalizable; E, experimental; D, direct; I, indirect; O, observational; PK, preschool; K, Kindergarten; 1-3, Grades 1-3. CELF-4, Clinical Evaluation of Language Fundamentals—Fourth Edition (Semel et al., 2003); TEGI, Test of Early Grammatical Impairment (Rice and Wexler, 2001); TROG-2, Test for Reception of Grammar—Second Edition (Bishop, 2003); TNL, Test of Narrative Language (Gillam and Pearson, 2004); PPVT, Peabody Picture Vocabulary Test (Dunn and Dunn, 2007); EVT-2, Expressive Vocabulary Test—Second Edition (Williams, 1997); GMRT, Gates McGinite Reading Test (Gates and McGinitie, 2000); WRMT, Woodcock Reading Mastery Test—Revised (Woodcock, 1998); WJ-III NU (Woodcock et al., 2001); KBIT, Kaufman Brief Intelligence Test—Second Edition (Kaufman and Kaufman, 2004); TOWRE, Test of Word Reading Efficiency (Torgesen et al., 2012); FAIR, Florida Assessment for Instruction in Reading (State of Florida Department of Education, 2009); TOPEL, Test of Preschool Early Literacy (Lonigan et al., 2007); CLASS, Classroom Assessment and Scoring System (Pianta et al., 2008); ISI, Individualizing Student Instruction Classroom Observation System (Connor et al., 2009a); CLOP, Classroom Literacy Observation Profile (Children's Learning Research Collaborative, 2008); CLEP, Classroom Literacy Environment Profile (Wolfersberger et al., 2004)*.

### Direct measures

Direct measures were those administered by research staff directly to child participants (in Table 5, these measures are indicated with a “D” in column 3). Only measures that were relevant to LARRC's major aims and that had a reasonable administration time (approximately 25 min or less) were directly administered to children. Many of the direct measures were standardized, psychometrically validated measures that have been used extensively in previous research (e.g., Peabody Picture Vocabulary Test; Dunn and Dunn, 2007; Gates-McGinitie Reading Comprehension Test; Gates and McGinitie, 2000). To maintain consistency across grades and to adhere to manageable administration times for children, a few of these measures had to be modified. For example, the Word Structure subtest of the Clinical Evaluation of Language Fundamentals—4th edition (CELF-4; Semel et al., 2003), developed for assessing grammar in children in grades K and higher, was also administered to PK children to preserve continuity. Administration was modified to include a discontinue rule (8 consecutive incorrect responses) to decrease the length of the assessment and potential frustration for these young children, and the psychometric properties of the subtest were rigorously examined to ensure validity and reliability of scores for this age group.

Some measures of listening and reading comprehension, the Qualitative Reading Inventory (QRI; Leslie and Caldwell, 2011), CELF-4 Understanding Spoken Paragraphs (Semel et al., 2003), and Test of Narrative Language (TNL; Gillam and Pearson, 2004), were also modified to ensure consistency across grades. For the CELF-4 Understanding Spoken Paragraphs and QRI (both Reading and Listening subtests), administration time, appropriateness of content, and consistency were improved by pre-selecting a small number of assessment items that were administered to all children in a given grade, rather than utilizing different start points based on age and continuing to administer multiple items until a discontinue rule was reached. New items had to be written for both measures to ensure appropriateness for the PK children and to ensure that all children received items based on both narrative and expository text. It should be noted that in order to differentiate our adaptation of the QRI from the original measure, we referred our modified versions as the Reading Comprehension Measure (RCM) and Listening Comprehension Measure (LCM). For the TNL, the only modifications were that children were asked to complete a retell (subtest 5) prior to answering comprehension questions about the same item and PK children also completed this task, despite its age range of 5-years and older.

The limited availability of established measures for certain study constructs necessitated development of additional custom, experimental measures to directly assess children's skills. Experimental measures were particularly necessary to assess higher-level language skills, including inferencing, comprehension monitoring, and knowledge of narrative structure. The five measures created to assess these constructs were developed by a LARRC researcher (Cain) with considerable expertise in these areas. Each measure was adapted from prototypes available in the literature (e.g., Nezworski et al., 1982; Stein and Glenn, 1982; Fitzgerald and Spiegel, 1983; Baker, 1984; Yuill and Joscelyne, 1988; Wechsler, 1992; Cain and Oakhill, 1999, 2006; Oakhill and Cain, 2012) that showed at least preliminary evidence of validity and reliability. Two additional experimental measures of working memory, the Non-word Repetition Task (created by LARRC researchers Hogan, Gray, and Brinkley) and Memory Updating Task (adapted by LARRC researchers Cain and Hogan from Belacchi et al., 2010), were similarly developed. All final measures were aligned with our guiding principles, reviewed by LARRC methodologists and statisticians, and subjected to rigorous psychometric testing and review. For the majority of modified measures, psychometric review led us to conclude that the measures were psychometrically sound. In the absence of adequate psychometric characteristics, the measure was no longer used in analyses.

### Indirect measures

In order to assess other constructs that were of interest but not of primary study relevance, we used indirect measures (in Table 5, these measures are indicated with an “I” in column 3). Indirect measures were surveys or questionnaires that were completed by the child's family or teacher. Children's caregivers and/or teachers reported information concerning children's behavior regulation skills, reading motivation, learning behaviors, sleep habits, and home environments, and also provided additional demographic and background information (e.g., age, race, socioeconomic status, disability status, English-language learner status). In addition, teachers completed indirect measures to report on their own demographics and backgrounds, along with a number of additional attributes such as their sense of self-efficacy and teaching beliefs. Using questionnaires, teachers self-reported many attributes of their schools and classrooms (e.g., half-day vs. full-day program, class enrollment, language(s) of instruction, curriculum).

### Observational measures

Observational measures allowed for our team to examine classroom-related constructs that may contribute to the language and reading skills of children (in Table 5, these measures are indicated with an “O” in column 3). We conducted annual live and/or videotaped classroom observations for the PK cohort of children only, as these are the only children who participated in the full 5 years of the study. Classroom observations were used to assess (a) the classroom literacy environment via the Classroom Literacy Observation Profile (CLOP) in PK and K, which was loosely adapted from the Early Language and Literacy Classroom Observation Toolkit (Smith and Dickinson, 2002)and the Classroom Literacy Environment Profile (CLEP; Wolfersberger et al., 2004) for G1-G3, (b) the amounts and types of language and literacy instruction provided via an adaptation of the Individualizing Student Instruction Classroom Observation System (ISI; Connor et al., 2009a), and (c) the quality of instructional support, emotional support, and classroom organization via the Classroom Assessment Scoring System (CLASS; Pianta et al., 2008). These observational data provide rich information about the learning contexts experienced by children.

### Lessons learned

As will be discussed in the next two steps, this large number of measures created a substantial need for consistent training of assessors (Step 6). In addition, our team had to be creative with respect to how these many assessments could be administered to children without fatigue, attrition, or scheduling difficulties (Step 7). We were also incredibly grateful to have our methodologists on board as investigators who could do some “heavy lifting” for our psychometric reviews. Our methodologists also helped to ensure that we were using the appropriate number of measures to capture a latent variable. Consistent with the recommendations in American Educational Research Association, American Psychological Association, and National Council on Measurement in Education (2014), the use of multiple measures enhances the construct validity of the underlying attribute being examined. Rather than rely on a single measure of an attribute, information derived on several related measures or indicators of that construct allows for recognition of normal variation of an indicator and a more defensible conclusion regarding the reliability of that construct (Hancock and Mueller, 2001). Each measure necessarily contributes to the overall assessment of the construct. Additionally, the inclusion of multiple measures strengthens our longitudinal component, and will allow us to assess and test for longitudinal invariance across the set of indicators for a construct, which is not possible if only a single measure is collected. Thus, we recommend including multiple measures of a construct where ever possible, and in particular for those constructs of primary relevance to the study. In our case these were measures relevant to language development (i.e., vocabulary, grammar, higher-level language skills) and reading comprehension.

## Step six: Train assessors and develop data collection procedures

Organization and standardization of assessor training and data collection was particularly important for the LARRC study given the large number of assessments and geographic dispersion of assessors across four states. A number of organizational steps were undertaken to ensure that the data collected by assessors was of the highest quality. First, all four data collection sites were assigned ownership of particular measures. Measures were systematically assigned based on the relevant expertise of site researchers (e.g., particular site researchers may have used certain measures for previous studies and thus would be able to provide appropriate guidance on the administration and scoring of those measures). The assigned LARRC site used project-wide standardized templates to prepare the administration protocol, scoring protocol, and training materials for the measure. For established measures, the content of these materials adhered to the measure's administration and scoring manual. All materials were reviewed by other LARRC researchers, including methodologists and statisticians, as a means of quality control. The site was also responsible for answering and documenting any questions on measure administration or scoring as these arose, and for post-scoring relevant measures (see Step 8). Second, efforts were made to deliver the majority of assessor training materials uniformly online (e.g., narrated PowerPoint presentations, video exemplars, and online quizzes) to ensure that all assessors received the same training. All training materials were uploaded to our centralized project website and thus accessible to trainers and assessors regardless of location. Third, measures were divided into 11 blocks of approximately equivalent administration time. This decision was practical in nature as it allowed for assessors to specialize in the administration of one or more blocks without training on an overwhelming number of measures.

### Assessor training

Assessors completed training on every measure within their assigned block(s). Assessors underwent comprehensive measurement training and in-lab observations to ensure consistent measurement administration and fidelity. They first completed Human Subjects Protections training provided by the Institutional Review Boards at their respective universities. Next they downloaded and viewed the LARRC General Field Assessor Training presentation, which was a narrated PowerPoint that provided an overview of LARRC policies and procedures regarding professionalism, the assessment environment, assessment in school contexts, establishment of rapport, provision of encouragement/feedback, and child assent, along with basics of assessment administration and recording. Subsequently assessors completed standardized training specific to each measure within their assigned block(s), each of which was organized as a separate, self-study module. All sites had research staff who went through the training first to become reliable. These reliable research staff members then participated in mock assessment administrations with all subsequent assessors.

### Training for direct measures

For the direct child measures, each training module began with a narrated PowerPoint presentation and administration video example. These materials provided an overview of the measure's purpose, administration, and scoring. Trainees then reviewed the administration and scoring protocols for the measure. Upon completion of these self-study activities, trainees took an online quiz. A process of self-study and quizzing repeated until a score of 100% was achieved. Next, trainees completed two mock administrations with a reliable research staff member at their respective universities. During these mock administrations, the research staff member responded to the trainee's administration of the measure using a standardized script and also provided feedback regarding administration. In addition, the research staff member scored the trainee's adherence to the administration and scoring protocols using a fidelity checklist designed for that measure. Fidelity of 90% or better was necessary for assessors to administer measures to children participating in the LARRC study. Psychometric testing indicated that these training procedures, as implemented in conjunction with data collection and scoring procedures described in steps 7 and 8 below, resulted in a project-wide dataset that was valid and reliable. For example, internal consistency as measured by Cronbach's (1951) alpha coefficients ranged from 0.80 to 0.88 across sites for the TNL and 0.69 to 0.82 for the LCM (see Table 6). Moreover, interrater reliability for these two select measures was 0.96 and 0.98, respectively.

**Table 6:** **Cronbach's alpha reliability information for one standardized and one experimental direct child measure**.

	**Test of narrative language**	**Listening comprehension measure**
**SITE**		
OSU	0.88	0.82
KU	0.8	0.69
UNL	0.83	0.74
ASU	0.86	0.77
Overall alpha	0.87	0.78
ICC	0.98	0.96

### Training for observational measures

Recall that LARRC employed four observational measures, defined above: CLOP, CLEP, CLASS, and ISI. Training modules differed slightly for these observational measures. All assessors received the Human Subjects Protections and LARRC general field assessor training. For the CLOP and CLEP measures, trainees were required to visit three classrooms with an experienced research staff member to establish inter-rater agreement on the CLOP (for PK and K classrooms) and/ or CLEP (for G1-G3 classrooms). Trainees and the experienced research staff member visited each of the classrooms together and spent 15 min individually completing the CLOP and/ or CLEP for each classroom. Following the three observations, agreement between the experienced research staff member and each trainee was calculated for each item of the CLOP and/ or CLEP. Trainees had to demonstrate 90% agreement with the experienced research staff member to complete classroom observations and the CLOP and/ or CLEP independently in the field.

Training on the two other classroom measures, the ISI (Connor et al., 2009a) Coding System and CLASS (Pianta et al., 2008), differed substantially. Both of these measures involved coding from video and both were assigned to the same LARRC university site whose researchers held the requisite expertise required for coding reliability. ISI coders also received training prior to beginning video observation coding. The training included reading of the ISI Coding Manual, watching a PowerPoint presentation, coding along with the trainer, and individual coding of previously master-coded videos. Coders were required to attain an intraclass correlation (ICC) of 0.70 to become a reliable ISI coder. To maintain reliability, on an annual basis coders were required to view an online PowerPoint refresher and to independently code 30–45 min portions of three master-coded videos. Again, coders were required to attain an ICC of 0.70 to maintain their status as a reliable ISI coder. In addition, coders met bimonthly to conduct a drift check to decrease potential threats to inter-rater reliability. Across all years of the LARRC study, all ISI coding was completed by staff at the assigned site to ensure consistency and high reliability.

For similar reasons, CLASS was also only initially coded by staff at this same site (i.e., in Years 1 and 2). All coders completed a training course on the CLASS system that was delivered by a CLASS-certified trainer. All coders passed the standard benchmark to be considered a CLASS-reliable coder, which is 80% scoring agreement with five master-coded observations (calculated as total agreement across all 10 scales of the CLASS across all five observations) and agreement on any particular CLASS dimension on at least two out of five of the master-coded observations. Agreement was defined as a score within one point of the master code. CLASS coders were required to complete a recertification test each year by coding five video observations and meeting the same benchmark as the initial test. During the coding of a cohort, coders met monthly to code and discuss an observation to prevent scoring drift.

In Years 3 through 5, study protocol was altered such that assessors across all sites were trained to use the CLASS. Specifically, as the PK cohort of children dispersed into more elementary school classrooms with greater numbers of peers who were not necessarily participating in the LARRC study, teachers were often reluctant to allow their classrooms to be videotaped. In cases in which videotaping was refused, teachers were given the option of participating in live, non-videotaped classroom observations during which the CLOP and/ or CLEP and CLASS could be completed. To complete the latter, assessors at each site were trained to complete the CLASS, with the certified CLASS trainer traveling to each LARRC university to deliver the training described above. Drift checks were completed by posting video segments to the project's secure website, which were coded and discussed by assessors at all sites. Videotaped observations continued to be coded by the assigned site following the same procedures described for Years 1 and 2. During each year of data collection and coding, appropriate steps were taken to ensure the reliability of all video coding.

### Lessons learned

The blocked administration of assessments led to substantial ease of assessor training and scheduling. It truly allowed for assessors to specialize in a smaller number of measures to administer. We believe that this improved the quality of their data collection, though we did not empirically measure that. For scheduling purposes, it was also useful to examine which blocks of measures a participant still needed and work with a smaller number of assessors who could administer that block. Flexibility when working across sites was imperative given that each site had to function as its own entity while still maintaining the integrity of the project and relevant protocols. For example, one site implemented Saturday data collection, whereas other sites went into schools only during the school week.

## Step seven: Commence data collection

Data collection occurred on an annual basis each spring and was conducted by the four LARRC sites who recruited participants. Similar to assessor training, most data collection procedures were standardized across the four universities. These included using the same measures, the same assessment window (i.e., time period during which data was collected), and the same administration and scoring procedures. Any procedural issues or discrepancies were reviewed weekly during a project director conference call and, if needed, were taken to the larger Study 1 committee for review and approval. This oversight and communication process, outlined in **Figure 2**, supported standardization across sites. Project-specific response forms were created using a commercially-available data electronic data capture system, which is described in Step 9. For published measures, permission was obtained to adapt the response forms to this format.

### Direct measures

All children were assessed annually during a 20-week window (January–May). As discussed, assessments were divided into 11 blocks. These blocks were approximately equivalent in terms of administration time and also structured to meet particular assessment needs. For instance, some assessments needed to be administered in a particular order (e.g., the Peabody Picture Vocabulary Test was administered immediately preceding the Expressive Vocabulary Test); others required audio-recording to ensure scoring accuracy (see Step 8) and these measures were grouped together. Measures within blocks were administered in a predetermined, set order. The ordering of administration blocks, however, was not set. This allowed sites flexibility in terms of logistics and scheduling and also helped to reduce any potential bias due to order effects. Response forms for each measure were prepopulated with the child's LARRC identification numbers and assessment block number. Each block was labeled with a cover sheet indicating the child's name, his or her teacher's name, and school. This cover page was removed and destroyed prior to data entry.

### Indirect measures

Caregivers completed two questionnaires. In year 1, caregivers first completed a brief screening questionnaire that accompanied the consent form. This screening questionnaire provided very basic information pertaining to children's eligibility for the study. In year 1 and each subsequent year of the study, caregivers also completed a more comprehensive questionnaire during each annual assessment window. This questionnaire included the indirect measures listed in Table 5. In a given year, the same questionnaire was completed by caregivers of all children, regardless of grade. The content of the questionnaire was reduced each year to decrease collection of redundant information.

Children's teachers completed three types of questionnaires. In year 1 of the study, teachers of consented children completed a screening questionnaire used to determine children's eligibility to participate in the study. In the spring of year 1 and each subsequent spring, teachers of enrolled participants completed a demographic and background questionnaire about themselves. This questionnaire included measures of teacher attributes listed in Table 5. Questions differed slightly for PK and elementary teachers. In the spring of year 1 and each subsequent spring, teachers also completed a questionnaire for each child in their classroom enrolled in the LARRC study. This questionnaire included the indirect behavioral regulation and reading motivation measures listed in Table 5.

### Observational measures

Videotaped and/or live classroom observations were conducted each year for children who started the study when they were in PK. Thus, this cohort had classroom observations for their PK, K, G1, G2, and G3 years. Observations in the PK year were completed when the classroom teacher was conducting “instructional time.” In subsequent grades, observations were completed during the classroom language and literacy block. All observations lasted a minimum of 80 min. As previously described, in Years 1 and 2 all observations were videotaped. Videotaped classroom observations followed procedures similar to those reported in the literature (Connor et al., 2009a,b; Piasta et al., 2009, 2014; Yeager Pelatti et al., 2014).

### Lessons learned

Three primary lessons came from the data collection process. First, it was imperative to have clear, consistent, and detailed training materials for administration and scoring of all measures. Second, these training materials were well-tested in advance of actual data collection. This ensured that we had checked for errors or inconsistencies across documents. Finally, we learned after year 1 to implement careful “recalibration” of all trained assessors to ensure that fidelity was maintained each year. In particular, measures that were more complicated to administer had several errors found only during data processing (Step 9). However, our strong communication system (Step 10) ensured that all project directors and assessors were immediately made aware of administration and scoring errors.

## Step 8: Develop a plan and team for data scoring

Given the multitude of data collected, LARRC utilized a commercially-available data capture system to by-pass hand entry/calculation and some of the human errors that may result from those processes. For certain measures, further scoring was required after collecting the data in the field. Additionally, certain measures required score calculation once scanned data was translated into the larger database. The details of these scoring processes are described below.

### Data scoring for post-scored measures

A few measures necessitated complex responses on the part of the child and subsequent scoring of children's verbal responses. For these measures, children's responses were audio-recorded and scored after collection in the field (post-scored) by trained research assistants. If a site was assigned to a post-scored measure, it was responsible for (a) development of all materials and protocols for post-scoring scoring and training of reliable coders, (b) completing post-scoring for relevant measures, and (c) calculating and reporting post-scoring reliability statistics. In the field, assessors completed administration forms (“pre-forms”) which sometimes included preliminary scoring. Audio and pre-form data from all sites were transferred to the owning site for post-scoring.

### Data scoring across all measures

As part of the data capture system, computer code was written to automatically calculate raw scores for each measure and allowed for scoring according to basal/ceiling rules provided in scoring manuals when applicable (e.g., the Test of Preschool Early Literacy has a ceiling rule of three incorrect responses in a row; Lonigan et al., 2007). Computer code was also written to automatically map raw scores to normative scores provided in scoring manuals (e.g., the Woodcock Reading Mastery Test provides normative scores; Woodcock, 1998). Scoring for established measures was determined according to instructions set forth in measure manuals. Scoring for experimental measures was determined through investigation of possible summary scores that would yield reliable results.

### Lessons learned

Throughout the study, several lessons were learned regarding the scoring of data. The first relates to post-scoring procedures. Post-scoring significantly complicated data processing, due primarily to the fact that two sets of forms were scanned and processed for each child. This sometimes led to errors in the database. In addition, post-scoring delayed the release of the data since it required more time for scoring and data cleaning. Two adjustments were made to post-scoring procedures throughout the study. First, a decision was made to discontinue post-scoring for less complex measures once it was determined that field assessors were skilled enough that their on-site scoring was accurate. For example, CELF Understanding Spoken Paragraphs was no longer post-scored after Year 2, and the RCM was no longer postscored after Year 4. Second, in Year 3 we realized that it was not essential for OSU to receive and process pre-forms. We, therefore, discontinued this procedure to minimize the possibility of error related to receiving two sets of data forms. There was a trade-off in that those forms were no longer accessed by and stored at the data hub, but the simplification in procedures was deemed worthwhile. The pre-forms were still sent to the owning site for use in post-scoring as necessary. In sum, simplification of procedures regarding number of forms associated with post-scoring was beneficial.

Another lesson learned relates to the use of missing data codes. It was important to have made clear *a priori* decisions about which types of information we wanted to capture and how that information would be coded. For example, we used -444 for measures that were not supposed to be administered at certain grades, -999 for items or cases that were missing for unknown reasons, and -333 for sum or standard scores that could not be calculated due to too many missing items. This allowed us to keep track of things like correct application of missing data rules and overall levels of unexpected missing data. Standardized missing data codes are therefore beneficial and should be utilized consistently throughout the study.

## Step nine: Develop and implement a plan for data management

The development and implementation of a strong data management plan was a critical component of the LARRC study. According to Burchinal and Neebe (2006), data management is a crucial component to preservation of data integrity between collection and analysis. The plan addressed personnel, technology, and data processing, all of which were centralized at one site (OSU), which served as the data hub.

### Personnel

Two teams were formed at OSU: a technology team and a data team. The technology team was responsible for the implementation of the electronic data capture and processing system, and the data team was responsible for all other aspects of data processing. In addition, teams of investigators and staff from across all five LARRC sites were formed to deal with measurement and data processing issues. These teams met regularly via conference calls.

### Technology

**Figure 1:**
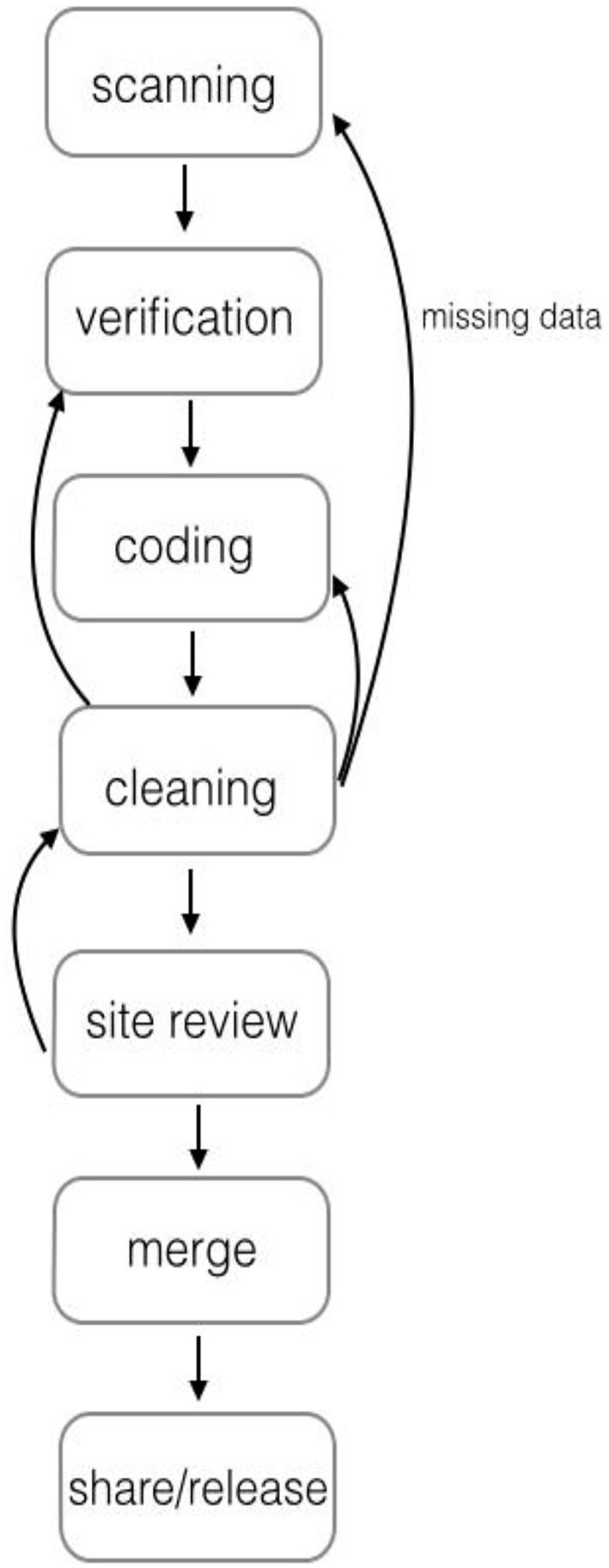
**The process by which data were sent to the primary site (OSU) for cleaning, coding, review, and release**. The process was iterative between the primary site and all other sites in order to obtain accurate identification number lists and ensure that any possible missing data were found prior to site review and use of data for analyses.

A commercially-available data capture system was chosen as the method for data collection and entry in order to reduce manual workload and to minimize data entry errors. Templates were created for each measure, and forms were then prepopulated with relevant information such as child identification number, birthdate, grade, teacher identification number, etc. The prepopulated forms were then made available to sites for download. Assessors completed these forms as they administered the measures, filling in the response bubbles to record child responses. Sites then scanned all forms to the data hub where they were processed. Figure 1 provides additional details.

### Data processing

Data processing involved intake/entry, cleaning, sharing, and merging of data. As previously described, data entry was conducted via each study site scanning electronic response forms to the data system at OSU. Once data were received, they were electronically processed and input to one cohesive database, at which point code was run to calculate scores.

In constructing the multi-site database, measures were investigated for degree of missingness (decisions regarding treatment of missing data are decided during analysis, and thus are not part of the data processing methods). Measures were deemed to have “acceptable” levels of missing data when data for less than 1% of the participants were missing for unknown reasons (vs. participants missing data due to incorrect score calculation). A cut point of 1% was determined to be acceptable as it is a minimal amount of missing data and allowed for a certain level of error, which is to be expected. Data for measures with acceptable levels of missing scores were then exported into a statistics software package. For measures with greater than 1% missing, sites were provided with lists of missing identification numbers and asked to verify and (re)scan those forms.

The next step in data processing was rigorous data cleaning. Datasets for every measure underwent a thorough, multi-step process of cleaning to reduce error as much as possible. Figure 1 provides the specific steps involved in this process. First, the electronic data system includes a step for verification of data in which any questionable items were checked (e.g., responses that were not filled in clearly on the response form). Next, a research assistant conducted a preliminary audit of the dataset, including random checks to ensure that data on the response forms matched what was in the dataset. Then the dataset received a more thorough cleaning by another member of the data team. Checks were conducted for errors at all steps in the process, including errors in test administration, completion of response forms (e.g., responses not filled in correctly or too faintly for the scanner to read it accurately), and coding. For example, checks were conducted for accuracy of score calculations (including total scores, subscales, and standard scores); accuracy of basal/ceiling rules; unexpected data patterns including missing items; use of correct missing data codes; examination of outliers; and additional comparisons between data on a random selection of the response forms and the dataset. When errors were found, the details were communicated to the technology team to be fixed. Then the next round of cleaning the dataset began. This iterative process continued until no errors were observed. A final check of each dataset was conducted by the owning site before it was approved for use.

Throughout data processing, detailed codebooks were created to serve four purposes: (a) provide information about the measures (e.g., reason for measure selection, description of the measure), (b) record reliability and validity details, (c) provide variable names and details (i.e., a data dictionary), and (d) track changes and decisions related to the measure and its data. Codebooks were prepared by the owning site following a standardized project-wide protocol and accessed by all investigators via the secured study Sharepoint. These codebooks are described further in Step 10.

### Lessons learned

Data management proved to be even more complex than anticipated. We continually refined and improved our processes throughout the study as we encountered problems or inefficiencies. We learned that the prepopulation of data forms with pertinent identifying information was essential for reducing error, and that this information should include the timepoint. Because this was a longitudinal study and data were collected across multiple sites, maintaining master ID lists and tracking changes in participants across timepoints was challenging. We learned to follow up with sites each year to verify and update the lists and we implemented a system for tracking participants who dropped from the study. Also related to monitoring activities across sites, we learned that assessment fidelity checks and communication about errors in test administration, discovered during data cleaning, were essential. If a particular type of administration error was discovered, this could be conveyed to sites and resolved prior to the next year of data collection.

## Step 10: Prepare for frequent and thorough communication, flexibility, and challenges

**Figure 2:**
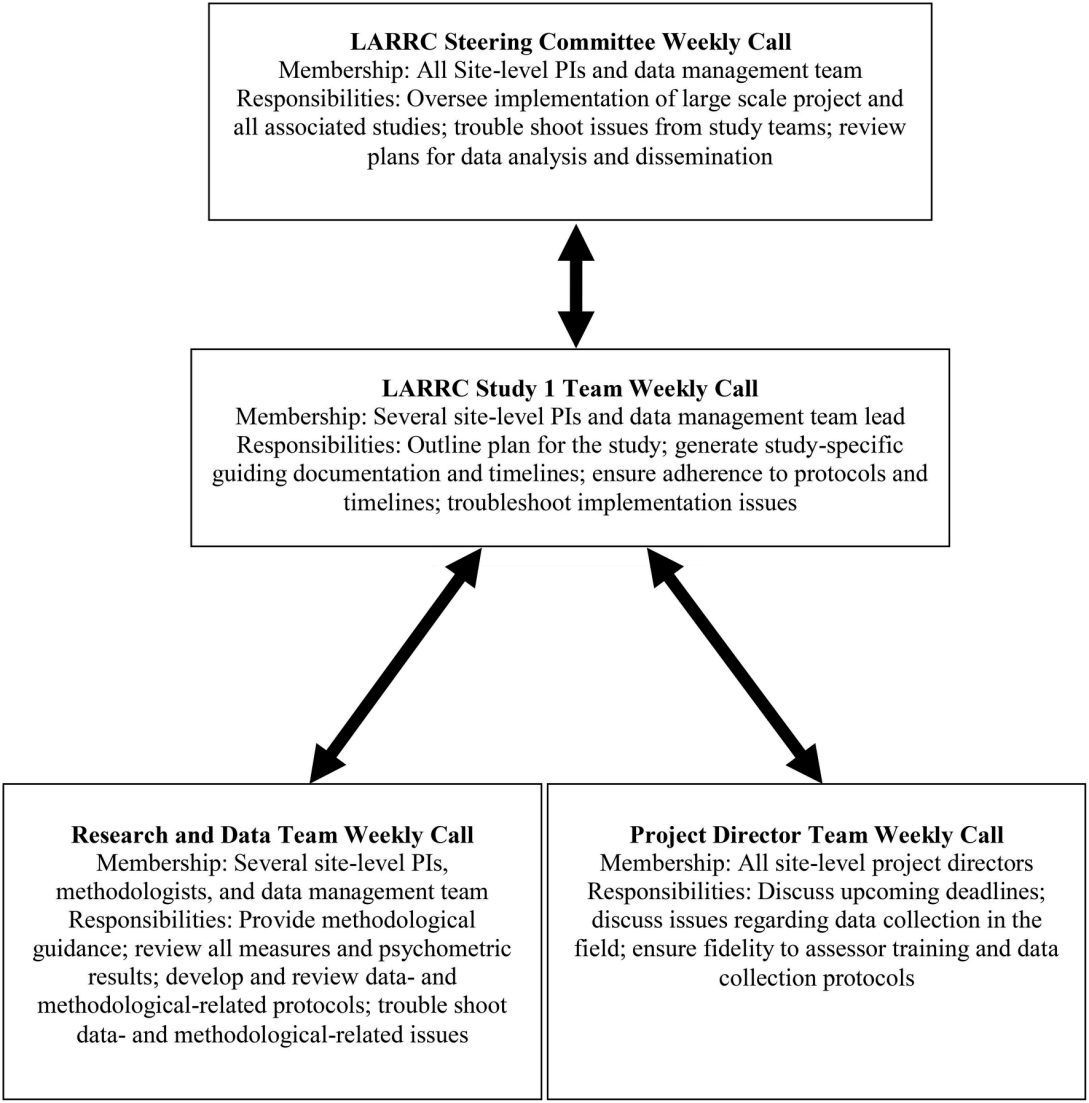
**Oversight and communication structure to support LARRC study 1 cross-site implementation**. Note that the data management team was comprised of both technology and data team members.

When designing and implementing the steps necessary to enact a longitudinal study, the LARRC team found it was imperative to prepare for frequent and thorough communication amongst partners, flexibility in decision making, and responsiveness to challenges. As mentioned above, teams of LARRC investigators implemented communication strategies through meeting regularly in person and several times weekly via conference call to discuss and make decisions regarding measurement and data processing issues (see Figure 2). In retrospect, this was considered by LARRC team members as one of the most crucial factors to the success of this project.

## Conclusion

In sum, multi-site, multi-grade longitudinal studies are complex endeavors. We believe that the enumerated steps, together with an appreciation for frequent communication and ability to flexibly respond to challenges, resulted in the successful execution of the LARRC study. Throughout the project, we capitalized on best research practices and the collective experiences and knowledge of our team to design and implement a large-scale study that met our goal of conducting rigorous research and ensuring data integrity across multiple field-based sites. Ultimately, because of the care and effort put into study design and execution, this work will contribute important empirical insights into how reading comprehension develops in the early grades, and some initial reports of findings are already available (see Language and Reading Research Consortium (LARRC), 2015a,c, in review). Through dissemination of our design strategies and articulation of lessons learned, we hope that research teams embarking on similar endeavors can be better equipped to anticipate the challenges of large-scale, multiple site project design, data collection, and management needs.

## Author note

This paper was prepared by a Task Force of the Language and Reading Research Consortium (LARRC) consisting of Kelly Farquharson (Convener), Kimberly A. Murphy, Ann O'Connell, Jill Pentimonti, and Shayne Piasta.

LARRC project sites and investigators are as follows:

**Ohio State University** (Columbus, OH): Laura M. Justice (Site PI), Richard Lomax, Ann O'Connell, Jill Pentimonti, Stephen A. Petrill^1^, Shayne B. Piasta.

**Arizona State University** (Tempe, AZ): Shelley Gray (Site PI), Maria Adelaida Restrepo.

**Lancaster University** (Lancaster, UK): Kate Cain (Site PI).

**University of Kansas** (Lawrence, KS): Hugh Catts^2^ (Site PI), Mindy Bridges, Diane Nielsen.

**University of Nebraska-Lincoln** (Lincoln, NE): Tiffany Hogan (Site PI), Jim Bovaird, J. Ron Nelson^3^.

**MGH Institute of Health Professions** (Boston, MA): Tiffany Hogan (Site PI)

^1^Stephen A Petrill was a LARRC co-investigator from 2010 to 2013.

^2^Hugh Catts is now at Florida State University.

^3^J. Ron Nelson was a LARRC co-investigator from 2010 to 2012.

## Author contributions

KF, corresponding author. Substantive contributions to the organization and structure of the entire paper. Specific writing contributions to “Steps 1–6.” LARRC, all PIs for the longitudinal project. Their roles included all details from basic design to data analysis. KM, substantive contributions to Steps 7–10 as well as data processing procedures.

## Funding

This work was supported by grant # R305F100002 of the Institute of Education Sciences' Reading for Understanding Initiative. We are deeply grateful to the numerous staff, research associates, school administrators, teachers, children, and families who participated. Key personnel at study sites include: Crystle Alonzo, Lisa Baldwin-Skinner, Garey Berry, Beau Bevens, Jennifer Bostic, Shara Brinkley, Janet Capps, Tracy Centanni, Beth Chandler, Lori Chleborad, Willa Cree, Dawn Davis, Jaclyn Dynia, Michel Eltschinger, Kelly Farquharson, Tamarine Foreman, Rashaun Geter, Abraham Aldaco Gastelum, Sara Gilliam, Miki Herman, Cindy Honnens, Elaine Joy, Trudy Kuo, Gustavo Lujan, Chi Luu, Junko Maekawa, Carol Mesa, Denise Meyer, Maria Moratto, Kimberly A. Murphy, Marcie Mutters, Amy Pratt, Trevor Rey, Amber Sherman, Shannon Tierney, and Stephanie Williams.

### Conflict of interest statement

The authors declare that the research was conducted in the absence of any commercial or financial relationships that could be construed as a potential conflict of interest. The views presented in this work do not represent those of the federal government, nor do they endorse any products or findings presented herein.

## Footnotes

^1^The PI at UNL subsequently moved to Massachusetts General Hospital—Institute of Health Professions. All activities related to the longitudinal portion of this project, as described here, continued to take place at UNL and remained under the direction of the original PI.

^2^Power analyses originally conducted for purposes of grant submission indicated 200 children in each of K through G3. In the first year of the study, further power analyses indicated that 120 children per grade was sufficient for achieving >80% power. As such, 120 children per grade were randomly selected to fully participate in all assessments conducted as part of the larger study. The 80 additional children recruited were retained as a secondary sample for the first two years of the study; these children participated in indirect assessments only and were used to replace any children who attrited from the general sample to preserve the sample size. In year 3, once attrition rates were established as being minimal, any children remaining in the secondary sample were thanked for their participation and no longer asked to complete any study measures.

## References

[B1] American Educational Research Association American Psychological Association, National Council on Measurement in Education. (2014). Standards for Educational and Psychological Testing. Washington, DC: American Educational Research Association.

[B2] Baker L. (1984). Children's effective use of multiple standards for evaluating their comprehension. J. Educ. Psychol. 76, 588–597. 10.1037/0022-0663.76.4.588

[B3] Belacchi C. Carretti B. Cornoldi C. (2010). The role of working memory and updating in coloured raven matrices performance in typically developing children. Eur. J. Cogn. Psychol. 22, 1010–1020. 10.1080/09541440903184617

[B4] Bishop D. V. (2003). Test for Reception of Grammar: TROG-2 version 2. Pearson Assessment.

[B5] Burchinal M. R. Neebe E. (2006). I. Data management: recommended practices. Monogr. Soc. Res. Child Dev. 71, 9–23. 10.1111/j.1540-5834.2006.00402.x

[B6] Cain K. Oakhill J. V. (1999). Inference making ability and its relation to comprehension failure in young children. Read. Writ. 11, 489–503. 10.1023/A:1008084120205

[B7] Cain K. Oakhill J. V. (2006). Profiles of children with specific reading comprehension difficulties. Br. J. Educ. Psychol. 76, 683–696. 10.1348/000709905X67610 17094880

[B8] Connor C. M. Morrison F. J. Fishman B. J. Ponitz C. C. Glasney S. Underwood P. S. (2009a). The isi classroom observation system: examining the literacy instruction provided to individual students. Educ. Res. 38, 85–99. 10.3102/0013189X09332373

[B9] Connor C. M. Piasta S. B. Glasney S. Schatschneider C. Fishman B. J. Underwood P.. (2009b). Individualizing student instruction precisely: effects of child-by-instruction interactions on first graders' literacy development. Child Dev. 80, 77–100. 10.1111/j.1467-8624.2008.01247.x 19236394 PMC2648136

[B10] Cronbach L. J. (1951). Coefficient alpha and the internal structure of tests. Psychometrika 16, 297–334.

[B11] Cutting L. E. Scarborough H. S. (2006). Prediction of reading comprehension: relative contributions of word recognition, language proficiency, and other cognitive skills can depend on how comprehension is measured. Sci. Stud. Read. 10, 277–299. 10.1207/s1532799xssr1003_5

[B12] Douglas K. M. Albro E. R. (2014). The progress and promise of the Reading for Understanding initiative. Educ. Psychol. Rev. 26, 341–355. 10.1007/s10648-014-9278-y

[B13] Dunn L. M. Dunn L. M. (2007). Peabody Picture Vocabulary Test, 4th Edn. Upper Saddle River, NJ: Pearson Education.

[B14] Fitzgerald J. Spiegel D. L. (1983). Enhancing children's reading comprehension through instruction in narrative structure. J. Lit. Res. 15, 1–17. 10.1080/10862968309547480

[B15] Gates A. I. McGinitie W. H. (2000). Gates-mcginitie Reading Tests, 4th Edn. Boston, MA: Riverside.

[B16] Gillam R. B. Pearson N. (2004). Test of Narrative Language. Austin, TX: Pro-Ed.

[B17] Gough P. B. Tunmer W. E. (1986). Decoding, reading, and reading disability. Rem. Spec. Educ. 7, 6–10. 10.1177/074193258600700104

[B18] Hancock G. R. Mueller R. O. (2001). Rethinking construct reliability within latent variable systems, in Structural Equation Modeling: Present and Future, eds Cudeck R. du Toit S. Sörborm D. (Lincolnwood, IL: Scientific Software International), 195–216.

[B19] Hoover W. A. Gough P. B. (1990). The simple view of reading. Read. Writ. 2, 127–160. 10.1007/BF00401799

[B20] IES (2009). Reading for understanding research initiative, in Paper Presented at the Institute of Education Sciences. Available online at: http://ies.ed.gov/ncer/projects/program.asp?ProgID=62

[B21] Joshi R. M. Aaron P. G. (2000). The component model of reading: simple view of reading made a little more complex. Read. Psychol. 21, 85–97. 10.1080/02702710050084428

[B22] Joshi R. M. Aaron P. G. (2012). Componential model of reading (cmr). J. Learn. Disabil. 45, 387–390. 10.1177/0022219411431240 22879651

[B23] Kaufman A. S. Kaufman N. L. (2004). Kaufman Brief Intelligence Test. John Wiley & Sons, Inc.

[B24] Kline R. B. (2004). Principles and Practice of Structural Equation Modeling, 2nd Edn. New York, NY: Guilford Press.

[B26] Language and Reading Research Consortium (LARRC) (2015a). Learning to read: should we keep things simple? Read. Res. Q. 1, 1–19.

[B27] Language and Reading Research Consortium (LARRC) (2015b). The dimensionality of Spanish in young spanish-english dual language learners. J. Speech Lang. Hear. Res. 58, 754–766. 10.1044/2015_JSLHR-L-13-0266 25787917

[B25] Language and Reading Research Consortium (LARRC) (2015c). The dimensionality of language ability in young children. Child Dev. 86, 1948–1965. 10.1111/cdev.12450 26509742

[B28] Leslie L. Caldwell J. S. (2011). Qualitative Reading Inventory, 5th Edn. Boston, MA: Pearson.

[B29] Lonigan C. J. Wagner R. K. Torgesen J. K. Rashotte C. A. (2007). Test of Preschool Early Literacy. Austin, TX: Pro-Ed.

[B30] Murphy K. Farquharson K. LARRC (in press). Investigating profiles of lexical quality in preschool and their contribution to first grade reading.

[B31] McCardle P. Scarborough H. S. Catts H. W. (2001). Predicting, explaining, and preventing children's reading difficulties. Learn. Disabil. Res. Pract. 16, 230–239. 10.1111/0938-8982.00023

[B32] McDonald S.-K. Keesler V. A. Kauffman N. J. Schneider B. (2006). Scaling-up exemplary interventions. Educ. Res. 35, 15–24. 10.3102/0013189X035003015

[B33] McNamara D. S. Magliano J. (2009). Toward a comprehensive model of comprehension, in The Psychology of Learning and Motivation, Vol. 51, ed Ross B. (Burlington, VT: Academic Press), 297–384.

[B34] Nezworski T. Stein N. L. Trabasso T. (1982). Story structure versus content in children's recall. J. Verbal Learn. Verbal Behav. 21, 196–206. 10.1016/S0022-5371(82)90561-8

[B35] National Research Council (2002). Neem: A Tree for Solving Global Problems. The Minerva Group, Inc.

[B36] Oakhill J. V. Cain K. (2012). The precursors of reading ability in young readers: evidence from a four-year longitudinal study. Sci. Stud. Read. 16, 91–121. 10.1080/10888438.2010.529219

[B37] Ong-Dean C. Huie Hofstetter C. Strick B. R. (2011). Challenges and dilemmas in implementing random assignment in educational research. Am. J. Eval. 32, 29–49. 10.1177/1098214010376532

[B38] Perfetti C. A. (2007). Reading ability: lexical quality to comprehension. Sci. Stud. Read. 11, 357–383. 10.1080/10888430701530730

[B39] Perfetti C. A. Landi N. Oakhill J. (2005). The acquisition of reading comprehension skill, in The Science of Reading: A Handbook, eds Snowling M. J. Hulme C. (Malden, MA: Blackwell), 227–247.

[B40] Pianta R. C. Karen M. Paro L. Hamre B. K. (2008). Classroom Assessment Scoring System (CLASS) Manual, Pre-K. Baltimore, MD: Paul H. Brookes Publishing Company.

[B41] Piasta S. B. Connor C. M. Fishman B. Morrison F. J. (2009). Teachers' knowledge of literacy, classroom practices, and student reading growth. Sci. Stud. Read. 13, 224–228. 10.1080/10888430902851364

[B42] Piasta S. B. Yeager Pelatti C. Miller H. L. (2014). Math and science learning opportunities in preschool classrooms. Early Educ. Dev. 25, 445–468. 10.1080/10409289.2013.817753 25489205 PMC4256529

[B43] Rice M. L. Wexler K. (2001). Rice/Wexler Test of Early Grammatical Impairment. Psychological Corporation.

[B44] Rudd A. Johnson R. B. (2008). Lessons learned from the use of randomized and quasi-experimental field designs for the evaluation of educational programs. Stud. Educ. Eval. 34, 180–188. 10.1016/j.stueduc.2008.08.002

[B45] Semel E. Wiig E. H. Secord W. A. (2003). Clinical Evaluation of Language Fundamentals, 4th Edn. Bloomington, MN: Pearson.

[B46] Smith M. W. Dickinson D. K. (2002). Early Language and Literacy Classroom Observation Toolkit, Research Edn. Baltimore, MD: Brookes.

[B47] State of Florida Department of Education (2009). Florida Assessment for Instruction in Reading (FAIR). Available online at: http://www.fcrr.org/FAIR/index.shtm

[B48] Stein N. L. Glenn C. G. (1982). Children's concept of time: the development of a story schema, in The Developmental Psychology of Time, ed Friedman W. J. (New York, NY: Academic), 260–282.

[B49] Sterba S. K. Christ S. L. Prinstein M. J. Nock M. K. (2011). Beyond treating complex sampling designs as simple random samples: data analysis and reporting, in Handbook of Ethics in Quantitative Methodology, eds Panter A. T. Sterba S. K. Panter A. T. Sterba S. K. (New York, NY: Routledge/Taylor & Francis Group), 267–291.

[B50] Torgesen J. K. Wagner R. Rashotte C. (2012). TOWRE-2 Test of Word Reading Efficiency. Austin, TX: Pro-Ed.

[B51] US Department of Education Institute for Educational Sciences [IES] (2013). Assessing Attrition Bias. Available from http://ies.ed.gov/ncee/wwc/documentsum.aspx?sid=243

[B52] Wagner E. H. Koepsell T. D. Anderman C. Cheadle A. Curry S. G. Psaty B. M.. (1991). The evaluation of the Henry J. Kaiser Family Foundation's community health promotion grant program: design. J. Clin. Epidemiol. 44, 685–699. 10.1016/0895-4356(91)90029-9 2066747

[B53] Wechsler D. (1992). Wechsler Intelligence Scale for Children, 3rd Edn. London: The Psychology Corporation.

[B54] Williams K. T. (1997). Expressive Vocabulary Test Second Edition (EVT™2). J. Am. Acad. Child Adolesc. Psychiatry 42, 864–872.

[B55] Woodcock R. W. (1998). Woodcock Reading Mastery Tests, Revised, Examiner's Manual. American Guidance Service.

[B56] Woodcock R. W. McGrew K. S. Mather N. (2001). Woodcock-Johnson Tests of Achievement. Itasca, IL: Riverside Publishing.

[B57] Wolfersberger M. E. Reutzel D. R. Sudweeks R. Fawson P. C. (2004). Developing and validating the classroom literacy environmental profile (clep): a tool for examining the “print richness” of early childhood and elementary classrooms. J. Lit. Res. 36, 211–272. 10.1207/s15548430jlr3602_4

[B58] Yeager Pelatti C. Piasta S. B. Justice L. M. O'Connell A. A. (2014). Language- and literacy-learning opportunities in early childhood classrooms: children's typical experiences and within-classroom variability. Early Child. Res. Q. 29, 445–456. 10.1016/j.ecresq.2014.05.004

[B59] Yuill N. Joscelyne T. (1988). Effect of organizational cues and strategies on good and poor comprehenders' story understanding. J. Educ. Psychol. 80, 152–158. 10.1037/0022-0663.80.2.152

